# Perspectives on an Intensive Hospital-Based Smoking Cessation Intervention in Relation to Transurethral Resection of the Bladder Tumour (TURBT): Interviews with Patients, Relatives, and Clinicians

**DOI:** 10.3390/ijerph22040555

**Published:** 2025-04-03

**Authors:** Line Noes Lydom, Rie Raffing, Susanne Vahr Lauridsen, Ingrid Egerod, Ulla Nordström Joensen, Hanne Tønnesen

**Affiliations:** 1 WHO-CC/Clinical Health Promotion Centre, The Parker Institute, Copenhagen University Hospital—Bispebjerg and Frederiksberg, 2000 Frederiksberg, Denmark; 2 Department of Surgery, Centre for Perioperative Optimization, Copenhagen University Hospital—Herlev and Gentofte, 2730 Herlev, Denmark; 3 Department of Clinical Medicine, University of Copenhagen, 2000 København N, Denmark; 4 Department of Urology, Copenhagen University Hospital—Herlev and Gentofte, 2730 Herlev, Denmark; 5 Department of Intensive Care, Copenhagen University Hospital—Rigshospitalet, 2100 København Ø, Denmark; 6 Department of Urology, Copenhagen University Hospital—Rigshospitalet, 2100 København Ø, Denmark

**Keywords:** smoking cessation intervention, transurethral resection of bladder tumours, TURBT, bladder cancer, multi-perspective interviews, GSP

## Abstract

Smoking is a major risk factor for bladder cancer and postoperative complications. Therefore, urological guidelines strongly recommend smoking cessation. Notwithstanding, many patients continue to smoke beyond the time of diagnosis. By using the qualitative methodology, this study aimed to explore barriers, facilitators, and recommendations related to the intensive smoking cessation Gold Standard Programme (GSP) from the multi-perspective view of patients treated with transurethral resection of the bladder tumour (TURBT), their relatives, and clinicians. We conducted semi-structured individual interviews with eight patients, four relatives, and six clinicians in the urology setting. Data were analysed using the Framework Method. All participants perceived the GSP positively. Across the three groups, five categories emerged describing barriers and facilitators: perceptions of the GSP, pragmatic factors, health-related factors, psychological factors, and relational and communicative factors. Similarly, recommendations were represented in two categories: the GSP and pragmatic factors. While facilitators were relatively similar across the three groups, barriers were dissimilar or contradictory. The clinicians expressed the most challenges related to relational and communicative factors. The patients mainly had recommendations related to the GSP, while the clinicians’ recommendations focused on pragmatic factors for conducting the GSP. The potential involvement of relatives needs to be further investigated.

## 1. Introduction

There is a well-established link between smoking and the development and aggravation of bladder cancer with approximately 50% of cases directly attributed to smoking [1]. Therefore, smoking cessation is strongly recommended in the European Association of Urologists’ treatment guidelines for bladder cancer [2]. Two recent reviews reported contradictory results on the impact of smoking cessation on recurrence after shorter follow-up [3,4]. Rink showed that at least ten years of smoking cessation significantly reduced both the recurrence and the progression [5]. However, they all recommend smoking cessation.

The imperative for smoking cessation in this patient group can be viewed as threefold: to reduce the risk of complications associated with future surgeries and other treatments [6], to reduce the likelihood of disease recurrence following treatment [3], and to prevent the development of other smoking-related conditions or diseases [7]. Nevertheless, many patients continue to smoke both at the time of diagnosis and afterwards [8].

Tobacco addiction is a complex condition influenced by various factors, including social and environmental influences, learned or conditioned behaviours, and physical dependency [9]. The decision to change a risky lifestyle is often shaped by the actions and influence of close connections, particularly relatives such as spouses or siblings [10]. Clinical staff play a key role in identifying smokers and referring these patients to available smoking cessation interventions (SCIs). Urologists play a pivotal role in advising newly diagnosed patients on the potential benefits of smoking cessation [11]. However, studies have highlighted barriers faced by clinicians in hospital settings, including limited knowledge of smoking cessation support, time constraints, and a perceived lack of patient motivation [12,13,14,15]. Therefore, understanding recommendations, facilitators, and barriers perceived by key individuals involved with the patient’s treatment pathway may inform the refinement and implementation of smoking cessation strategies targeted to this patient population.

We have recently shown that nearly one third of patients were still smoking at the time of transurethral resection of the bladder tumour (TURBT), which was performed for diagnosis, complete resection, or follow-up for cancer recurrence [16]. In addition, we conducted a randomised trial in this patient population, demonstrating that smoking cessation was more effective in the surgical setting than standard practice (outpatient municipal setting). By the end of the intervention, 37% of participants in the hospital-based group reported successful quitting compared to 6% in the standard practice group (*p* = 0.04) [17]. The present study was planned as a nested interview study to this RCT [18].

The smoking cessation intervention in both settings utilised the intensive six-week Danish Gold Standard Programme (GSP), which includes weekly counselling, patient education, motivational support, and nicotine replacement therapy (NRT) [19]. In the surgical urology setting, trained smoking cessation counsellors who were also experienced nurses specialising in urology or oncology but not directly involved in patient treatment provided the GSP.

The standard treatment group received brief smoking cessation advice and was referred to the GSP, which was provided free of charge in the municipality in accordance with recommendations for treating smokers in Danish hospitals [20]. The intervention group participated in the GSP provided within the surgical urology setting.

By using qualitative methodology, this study aimed to explore barriers, facilitators, and recommendations related to the intensive smoking cessation Gold Standard Programme (GSP) from the multi-perspective view of patients treated with transurethral resection of the bladder tumour (TURBT), their relatives, and clinicians.

## 2. Materials and Methods

To address the aim, we found that qualitative methodology was the most suitable design to gain insight into human experiences, attitudes, and behaviour. We conducted a qualitative descriptive study using the Framework Method [21,22,23], as this method is helpful for comparing and contrasting data during within-case and across-case analysis [24].

The study was conducted and reported in compliance with the consolidated criteria for reporting qualitative studies (COREQ) checklist [25]. It was approved by the Danish Scientific Ethical Committee (H-20081571) and The Danish Data Protection Agency (P-2020-95) as a part of the STRONG for Surgery, Strong for Life study [26]. All participants received oral and written information about the study before deciding whether to participate. Written informed consent was obtained from all included participants.

### 2.1. Recruitment and Sampling Strategy

We aimed to follow the principle of data saturation to determine the number of included participants. A criterion sampling method was employed to include patients who had completed the GSP within the surgical urology setting. Relatives were recruited through a convenience sampling strategy with patients asked to nominate family members, partners, or close friends to participate in the study [27]. Clinicians, including both nurses and physicians from the Department of Urology, were purposively sampled based on their direct patient contact and close involvement in the TURBT treatment pathway rather than their seniority or age. The reason was that the perspectives of the ones interacting with the patients daily would be pivotal for a comprehensive investigation of the barriers, facilitators, and recommendations related to the GSP [27]. Since the GSP was provided by study nurses who were not involved in the TURBT treatment pathway, the interviewed clinicians did not have direct experience with delivering the GSP themselves. Participants were approached face to face (patients and clinicians) or by telephone (relatives).

### 2.2. Participants and Setting

Patients who completed the GSP intensive smoking cessation intervention in the surgical urology setting were eligible for the interview study. Adult relatives nominated by the participating patients were also eligible. Clinicians who were involved in the treatment pathway of patients undergoing TURBT were also eligible.

Patient inclusion was restricted to those who completed the GSP. The number of eligible patients who could nominate a close relative for participation limited the inclusion of relatives. The number of included clinicians was determined through data saturation based on a discussion between the researchers (LNL and RR) [27], which is a widely used approach in qualitative research to ensure comprehensive data collection until no new themes emerge.

### 2.3. Data Collection

An interview guide was developed based on the study’s aim to explore experiences, barriers, facilitators, and recommendations related to the GSP (Table 1). A semi-structured interview strategy was employed, allowing for the flexible exploration of specific topics while maintaining a standardised format [28]. To ensure the interview guide was comprehensive, acceptable, and sensitive to participants, a patient panel [29] from the STRONG for Surgery, Strong for Life study [26] was consulted during its development. The interview guide was also pilot tested with an individual who had participated in an intensive smoking cessation intervention (unrelated to TURBT treatment), leading to minor revisions of wording.

Two experienced female interviewers (LNL and RR) conducted the interviews. LNL is a nurse and PhD student, and RR is an anthropologist and PhD student who is experienced in qualitative research methods. LNL was involved in providing the GSP in the surgical urology setting and, therefore, knew the patients and clinicians beforehand, whereas RR did not. Both interviewers (LNL and RR) were present at all interviews with patients and relatives. Individual interviews with clinicians were conducted by LNL only.

### 2.4. Trustworthiness

To foster self-awareness about the researchers’ impact and potential sources of bias during the research process [30], both interviewers were interviewed by an experienced researcher not otherwise involved with the study. These interviews explored areas such as their beliefs, previous experiences with the research subject, motivations for conducting the study, and qualifications for carrying out the research. Throughout the research process, LNL and RR discussed insights developed during the interviews and emerging themes across participants.

### 2.5. Data Analysis

Interviews were audio recorded and transcribed verbatim by LNL. During transcription, data were pseudo-anonymised and stored securely. Data were analysed using the Framework Method [21,22,23], which was supported by NVivo 14 software^®^ [31]. We applied a multi-perspective view in the final phase of the analysis to gain a richer understanding of needs, experiences, barriers, and facilitators by triangulation [32], which enabled us to explore complimentary and contradictory perspectives [21,22,23].

The analytical process was initiated with the familiarisation phase, during which the interview transcript was read several times. Data were then coded using a combined deductive and inductive approach [22,23]. The deductive approach applied predefined codes based on a priori issues introduced in the interview guide, while the inductive approach allowed for additional codes to emerge during the analysis. LNL coded the first three interviews, one from each participant group, forming the basis for the initial framework.

RR then coded the same three interviews, and the codes were compared with revisions made to the framework as needed. LNL coded the remaining interviews, while RR also coded one additional interview from the patient and relative group and all six interviews from the clinicians. These codes were also compared.

Subsequently, the coded text was condensed for each participant, resulting in a matrix of participants (patients, relatives, and clinicians) summarising each participant’s responses within each code. Additionally, a summary of the condensed content was created across participants in each group.

**Figure 1:**
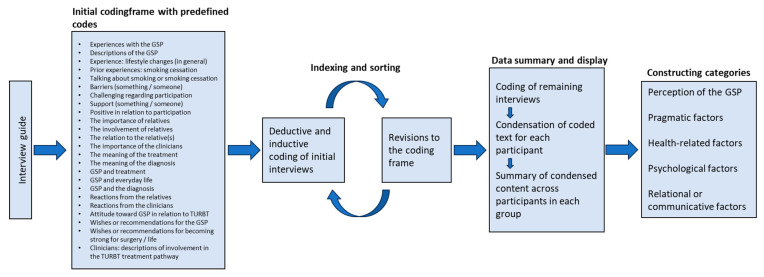
Data analysis process [21,22,23].

Finally, the condensed descriptions were compiled across the three groups into three matrices detailing barriers, facilitators, and recommendations with the content divided into categories and sub-categories (Figure 1).

## 3. Results

We interviewed 18 participants; see Table 2 for participant characteristics. Eight patients were invited to participate, all of whom accepted. Five patients were able to nominate relatives with one relative declining participation. Eight clinicians were invited; four nurses and two physicians participated, while two were unable due to time constraints.

The interviews were conducted between May 2022 and February 2024, and they took place either at the Urology department, the Parker Institute, or online for those who were unable to attend in person. Four patients had quit smoking at the time of the interview.

One relative had attended one GSP meeting, while three had no personal experience with the programme. Their relationship to the participants was as follows: one sibling, two children and one grandchild. Only one relative lived with the patient.

The clinicians were not aware of whether they had interacted with patients who participated in the GSP and had no direct experience with the programme. Therefore, the clinician interviews reflect attitudes towards the GSP or experiences from talking about smoking or smoking cessation in general with patients undergoing urology surgery rather than direct experience with the programme or indirect experiences through interactions with patients who had undergone the GSP.

### 3.1. Categories and Sub-Categories

We identified five categories describing the barriers and facilitators towards the GSP in the surgical urology setting: perceptions of the GSP, pragmatic factors, health-related factors, psychological factors, and relational or communicative factors. Sub-categories were grouped to visualise the complimentary and contradictory perspectives (Table 3). Recommendations were categorised into two groups: those for the GSP and those for pragmatic factors (Table 4).

#### 3.1.1. Perceptions of the GSP

All patients expressed satisfaction with being offered SCI in connection with TURBT, finding the programme both timely and relevant.

*“When they had to take a biopsy for the third time, I started to think that if it keeps looking like cancer, I should probably start considering it more seriously. I had this feeling that one day it might not just look like it—maybe it will be cancer. So, in a way, everything lined up quite well”*

(Patient 3)

Relatives also supported the idea of offering SCI alongside treatment, especially appreciating the immediate availability at the hospital and the absence of a waiting period.

This positive perception was echoed by the clinicians, who unanimously agreed that providing smoking cessation support to patients undergoing TURBT is beneficial, as quitting smoking before surgery is an advantage.

Patients highlighted the benefits of the structured counselling sessions, emphasising their clarity and progression. Measurements of carbon monoxide were valued as tangible indicators of progress, reinforcing motivation even before the physical benefits of cessation were felt. The counsellor relationship provided support, and participation in the programme fostered a sense of commitment, making it easier to adhere to smoking cessation goals.

*“It’s been good because you follow those steps where you say, “this time we’ll talk about this,” and I find that quite nice. The way it’s divided up—so now we’ve reached this point, and then we’ve reached the next point, and so on—is helpful”*

(Patient 3)

A few relatives and clinicians contributed to a minor degree with attitudes towards the SCI. No barriers were identified within this category.

Apart from the widely shared recommendation to offer SCI in connection with TURBT treatment, it was primarily the patients who provided specific recommendations for the GSP. These included adapting the GSP to align with the patient’s treatment journey and symptom burden, placing greater emphasis on patient education on the link between smoking and bladder cancer, and highlighting the rapid health benefits of smoking cessation. Additionally, patients recommended extending the period of complimentary NRT and making participation in SCI mandatory in relation to TURBT. Relatives contributed with one recommendation: to actively involve the relatives to let them share their pride in the patient’s efforts.

#### 3.1.2. Pragmatic Factors

Some of the patients experienced logistical factors, such as distance and parking, as barriers to participation despite successfully attending all meetings. On the other hand, clinicians expressed concerns that distance and travel time could hinder participation.

The experience that the meetings were integrated with other hospital appointments whenever possible made it easier for patients to attend. Clinicians also perceived the integration of the SCI with clinical treatment as a facilitator for participation. For patients, offering flexible timing and locations for meetings fostered a sense of control and improved engagement.

*“It worked well because I was already at the hospital for treatment, so I could see the smoking cessation counsellor before or after, usually before, then I’d go for my treatment and head home, so it fit in perfectly.”*

(Patient 1)

Clinicians cited constraints within the TURBT treatment pathway, such as brief patient interactions and time pressures, as reasons for smoking cessation discussions being overlooked or lacking depth.

*“… these patients sometimes don’t interact with a nurse at any point. So, discussing smoking cessation or lifestyle in general with them isn’t something, in my opinion, we’re particularly good at with this type of patient.”*

(Clinician 1)

The clinicians proposed several strategies to overcome the perceived barriers to implementing smoking cessation or discussions about smoking in clinical practice. These included offering flexible meeting options, such as meetings via video link, and incorporating nudging through the electronic medical records to prompt clinicians to address smoking cessation or refer patients to relevant programmes. Additionally, they recommended streamlining the referral process to smoking cessation interventions and providing training to enhance clinicians’ skills in discussing lifestyle changes.

#### 3.1.3. Health-Related Factors

The physical addiction to nicotine was identified as a significant barrier to cessation by all three groups. Patients and their relatives highlighted NRT as a key facilitator. Providing free NRT during the study period was particularly important for some participants, as it alleviated the financial burden associated with cessation support. Surprisingly, the clinicians did not mention the use or advantages of NRT.

Across all three groups, the motivational impact of recognising the serious health effects of smoking, especially in relation to their personal circumstances, was highlighted. Clinicians emphasised their crucial role in raising awareness about the risks of smoking and the importance of smoking cessation.

*“I think it’s simply that (the patient) was frightened by the diagnosis. There is strong evidence that it’s due to smoking and that quitting can help, so that’s most likely why (the patient) stopped.”*

(Relative 2)

The significance of the timing of the offer was highlighted. Patients described how the combination of health issues and the offer of support could raise a sense of openness and a strong motivation to change their behaviour. Some described how they welcomed the offer of the GSP as they felt ready to engage. While others, feeling overwhelmed by emerging health issues after the programme’s initiation, experienced barriers to continued participation or full engagement.

*“And there was a pause in my smoking. Unfortunately, when I experienced a lot of pain after the radiation therapy, I ended up starting again, and I haven’t entirely stopped yet”*

(Patient 1)

#### 3.1.4. Psychological Factors

Both relatives and clinicians and, to a minor degree, the patients described smoking as a source of comfort during difficult times in an illness trajectory. This reliance on smoking as a coping mechanism was perceived as a major barrier to cessation. Considering this, the timing of the SCI was identified as a possible challenge by the clinicians, as it may not align with the patient’s readiness to engage.

*“Smoking is also a form of comfort, making it difficult to be told that they are seriously ill and will have to go through a lot, and then, on top of that, being told they’re not allowed to do the one thing that helps ease their anxiety”*

(Clinician 4)

Another significant barrier from the clinicians’ perspective is a presumed lack of motivation for smoking cessation among the patients. However, some clinicians express that providing information about smoking cessation and available support, even when the patients are not currently ready to change their habits, may support the development of motivation in the long term. The importance of motivation is mirrored by the patients emphasising that the will to quit is the most important driver for successful cessation efforts with NRT and the counselling sessions as valuable support.

#### 3.1.5. Relational or Communicative Factors

All groups identified support from relatives as a facilitating factor. Conversely, lack of support, such as continued smoking by the relative, was perceived as a barrier by the clinicians. Meanwhile, the patients noted that not having any relatives to offer support could also pose a challenge.

Patients expressed that the clinicians’ recognition of the smoking cessation effort was valued. However, clinicians voiced concerns about damaging their relationship with the patient by appearing paternalistic or due to the sensitive nature of the topic, which they saw as a barrier to discussing smoking. Clinicians also noted that most patients are already aware of the harmful effects of smoking and felt that providing further information on the subject might be redundant. Nonetheless, they recognised that while there may exist a general awareness of the harms of smoking, the patients may lack knowledge of the specific risks associated with bladder cancer diagnosis and treatment. Patients, relatives and clinicians all mentioned that information about the link between bladder cancer is an important motivating factor.

The clinicians described an unstructured approach to talking to patients about smoking in the clinical setting, where the address of the issue may depend on having a good relationship with the patient or the patient themselves initiating the conversation. The noticeable smell of cigarette smoke was mentioned as a facilitator of conversations about smoking.

*“Well, I don’t find that there is any common practice where smoking cessation is routinely discussed with patients. It’s very individual, and I think it largely depends on, well, what kind of relationship you have with this particular type of patient or if the patients themselves ask about it.”*

(Clinician 1)

## 4. Discussion

Across the groups of participants, all viewed the offer of a smoking cessation intervention in relation to TURBT treatment as both timely and relevant. We identified however, both complimentary and contradictory perspectives.

This is the first study to examine the barriers, facilitators, and recommendations to an ongoing smoking cessation intervention in the surgical urology setting from the multi-perspective view of the patients, their relatives, and clinicians. A previous study investigated barriers and facilitators to smoking cessation from all three perspectives up to more than 18 months after other cancer diagnoses. Amongst others, both patients and relatives lack meaningful discussions about smoking or referral as well as the benefits of smoking cessation. In addition, they call for hospital-based smoking cessation intervention to be integrated into routine hospital attendance [33], which is closely related to the aim and setting of our study, and this was further supported by the results of another interview study, showing that the patients experienced preoperative smoking and alcohol intervention as an integrated part of the surgical treatment [34]. Other studies have investigated the perspectives of two of the groups: patients and clinicians [35,36,37,38] or patients and relatives [39,40].

The three participant groups expressed similar categories and almost similar groups of sub-categories as facilitators. Noticeably, both the patients and relatives mentioned NRT as an important support during smoking cessation, while NRT was not mentioned by the clinicians. This is surprising considering the robust evidence for the effectiveness of NRT in supporting smoking cessation [41]. Nevertheless, the broad platform of understanding and experiencing similar facilitators would ease the implementation of smoking cessation intervention among this patient group as recommended in international guidelines [2].

While all were positive and no barriers were expressed in relation to the GSP in the perioperative period, many other barriers were expressed, which were mostly voiced by the clinicians. They included the brief contact with the patients, an unstructured approach to conversations about smoking in relation to bladder cancer diagnosis, treatment and surgery, lack of time and lack of knowledge, which have frequently been expressed by clinicians in other studies as well [12,13,14,35]. Interestingly, they were not mentioned by the patients or the relatives in our study.

The perceived lack of patient motivation was identified as a major barrier among the clinicians in our study, which is a perspective that aligns with findings from other studies [13,14]. Again, this concern was not raised among the patients or relatives, but patients did express that their own will to quit was a major facilitator for smoking cessation.

Lack of support from relatives was identified as a barrier, while support was recognised as a facilitator by all groups. Similar findings have been reported in a study on smoking cessation intervention for patients scheduled for surgery and their relatives [39].

In our study, the clinicians mentioned factors such as smoking being a sensitive topic to address or the risk of damaging the patient–clinician relationship. This was not mentioned by either patients or relatives. On the contrary, the patients mentioned that they would appreciate recognition of their smoking cessation efforts by the clinicians. Similar to our findings, Wells et al. [33] found that clinicians often feel uneasy discussing smoking with patients, partly due to the perceived sensitive nature of the topic, whereas the patients generally expect the issue to be brought up during consultations [33]. This is in line with findings from other studies [35,36,38]. This reluctance among clinicians was also highlighted in a systematic review of clinician-reported barriers to smoking cessation in hospital settings [13]. Among others, a Danish study has previously shown that smoking among clinicians reduces their counselling on smoking cessation among patients [42]. This would hardly be the case in our study, as only one of the six clinicians was a current smoker.

Another barrier mentioned by the clinicians was the lack of a structured approach to discussing smoking in the clinical context. An example of this was the smell of smoke, which by some was described as a facilitator for initiating conversations about smoking with patients. The smell of smoke as a trigger for such discussions was also highlighted by clinicians in another study [38].

There is a major discrepancy between the clinicians and the patients regarding the perceived barriers to smoking cessation. Our findings highlight the need to integrate smoking cessation into clinician training to ensure that patients undergoing bladder cancer treatment receive smoking cessation interventions in accordance with recommendations in both national and European guidelines [2,43]. Providing all patients with access to effective smoking cessation interventions will help optimise treatment outcomes for bladder cancer while reducing disparities in outcomes among those who are still smoking at the time of diagnosis [44].

Interestingly, all patients, relatives and clinicians expressed positive attitudes towards providing intensive SCI in connection with TURBT. However, recommendations were mentioned less frequently than facilitators and barriers. We identified a difference between patients and clinicians regarding recommendations. Patients had the most wishes for the programme, while the clinicians primarily proposed solutions to mitigate perceived barriers. This difference seems natural, as patients are the only group with direct experience of the GSP.

Overall, the triangulation [45] used in this study has illustrated interesting differences between the clinicians and the patients regarding barriers and recommendations in relation to smoking cessation in the hospital setting. These are important to address to successfully support the patients in their effort to obtain the best possible health and disease management in the short and long term. The relatives often agree with the patients and the common facilitators, both of which describe a useful platform on which to build further.

A strength of this study was its thorough analytical approach to multi-perspective examination, incorporating views from the patients, their relatives, and clinicians. This approach enhances the understanding of the GSP in the surgical urology setting by highlighting both complimentary and contradictory perspectives among the three groups. Another strength of this study was the presence of two interviewers during most interviews. This arrangement facilitated deeper probing, as one interviewer concentrated on conducting the interview while the other focused on active listening and posing supplementary questions. Furthermore, the conduction of a pre-interview of the interviewers increased the trustworthiness.

However, this study also has its limitations. Firstly, all participating patients were recruited from those who had completed the full GSP within the surgical urology setting. As a result, they may represent a more motivated group compared to patients who declined to participate in the GSP or those who started but did not complete the SCI. This may limit the study’s ability to capture the perceived barriers to the GSP fully. Another bias is the small number of relatives who participated with only one partially living with the patient. The study could have been strengthened by including more relatives, particularly those who were partners and co-habiting with the patients, as this could have provided further insights into the support dynamics [8]. However, including more relatives was not possible. The presence of two interviewers during interviews with patients and relatives may introduce a bias. To mitigate this, a semi-structured interview guide was used to maintain consistency. On the other hand, this may allow for a more in-depth exploration of responses, with RR bringing an external perspective, while LNL had contextual knowledge of the clinical setting, which could be considered a strength. The presence of interviewer LNL, who was involved in providing the GSP and familiar with clinical staff, may have introduced bias. This could result in information or social desirability bias with interviewees potentially hesitant to express negative views of the GSP. Finally, the differing perspectives among the three groups, with patients having direct experience of the GSP, relatives having mainly indirect experience through conversations with the patient, and clinicians having no experience of the GSP, challenges the comparability of the knowledge that has emerged. However, since clinicians are expected to advise on smoking and refer patients to smoking cessation intervention, we believe that incorporating the clinician’s perspective provides important insights into the context in which smoking cessation interventions take place in practice. Our study included six clinicians. While no senior urologists were included, the perspective of these clinicians was relevant for understanding smoking cessation considerations within the bladder cancer treatment pathway. Future research could integrate senior urologists to explore additional perspectives on smoking cessation programmes in urological care.

The transferability of the results should be considered carefully. The conduction of the study may have introduced selection bias as the participants were recruited from a single centre at a university hospital in a Scandinavian high-income country with no financial charge for healthcare appointments in the public hospital system. This could limit the translation to other settings, cultures, and groups of participants. It would have been preferable to conduct the study in several centres to reduce the impact of the selection bias. However, to minimise selection bias, we applied an inclusive recruitment strategy, allowing all eligible patients who had completed the hospital-based GSP to participate, ensuring that a range of perspectives was included. In addition, we applied a multi-perspective approach by including patients, their relatives, and clinicians to ensure that we captured diverse viewpoints. While our findings may not be directly transferable to all settings, they provide valuable insights and inspiration for future studies.

### Perspectives

A successful smoking cessation intervention targeting surgical patients with smoking-related cancer could improve health outcomes and reduce the risk of disease recurrence. This is also of importance for the well-being of the relatives, as cancer disease and smoking affect the whole family, including through exposure to second-hand smoke.

To effectively support patients in successfully quitting, clinicians, specifically the healthcare system, generally need to build competencies. This would optimise the patient treatment pathway, improve outcomes, and lower the burden of cancer disease and other smoking-related comorbidities.

Society at large would benefit from less bladder cancer recurrence, fewer smokers, and healthier patients, thus allowing more persons back to work and less costs spent on healthcare.

The findings of this study call for implementation science to be involved in increasing clinical competencies in supporting patients in smoking cessation and in reducing the barriers to making it work in daily life. Another focus should be on evaluating the involvement and supportive role of the relatives.

## 5. Conclusions

The multi-perspective analysis in this qualitative study showed that all the participants positively perceived the GSP provided in the surgical urology setting. While facilitators were relatively similar across the three groups, barriers were dissimilar or contradictory. The clinicians expressed the most challenges related to relational and communicative factors. The patients mainly had recommendations related to the GSP, while the clinicians’ recommendations focused on pragmatic factors for conducting the GSP. Involving relatives may increase support for patients, but this needs to be explored further.

## Figures and Tables

**Table 1:** Structure of the interview guide.

Structure	Interview Topics
Introductory questions (icebreakers)	Can you describe the treatment you have undergone for your bladder disease? Please tell me about previous experiences with lifestyle change.
Transition question	How would you describe the programme to someone who has never heard of it before?
Main topics addressing the essence of the research questions.	The experience of participating in the programme. Recommendations, barriers, and facilitators regarding the programme. The context for lifestyle change. Changing lifestyle in connection with a treatment process. Strong for surgery—strong for life?
Final questions	Is there anything important to you that we have not discussed? Do you have any questions for us? Lastly, I would like to ask what you are taking away from this conversation?

**Table 2:** Characteristics of the participants.

		Patients (*n* = 8)	Relatives (*n* = 4)	Clinicians (*n* = 6)
**Male**		5	1	1
**Female**		3	3	5
**Median [range] age**		60 [52–77]	32 [26–55]	35 [27–48]
**Living alone**		6	N/A	N/A
**Smoking status**				
	Smoker	4	3	1
	Ex-smoker	4 *	1	1
	Never smoker	0	0	4

N/A: Not available. * Quitters after completion of the GSP.

**Table 3:** Categories and sub-categories for barriers and facilitators. Patients have directly experienced the GSP, while relatives have encountered it second-hand. Clinicians, meanwhile, expressed their attitudes and expectations regarding the patient experience.

	Barriers				Facilitators			
Categories	Sub-Categories	Patients	Relatives	Clinicians	Sub-Categories	Patients	Relatives	Clinicians
Perceptions of the GSP					The structure and content of the meetings	**X**		
					CO measurements	**X**		**X**
					Support from the smoking cessation counsellor	**X**	**X**	**X**
					The obligation towards the programme	**X**		**X**
Pragmatic factors	Transport and daily life	**X**		**X**	Integration of meetings with clinical treatment	**X**	**X**	**X**
					Flexibility and involvement	**X**		
	Brief contacts and time pressure in the clinic			**X**				
Health-related factors	The nicotine addiction	**X**	**X**	**X**	NRT	**X**	**X**	
	Disease and health complications	**X**	**X**		Health-related motivation	**X**	**X**	**X**
					The “right” timing	**X**	**X**	
Psychological factors	Smoking as a coping mechanism	**X**	**X**	**X**				
	Lack of patient motivation			**X**	The will to quit	**X**	**X**	
					It may plant a seed	**X**		**X**
Relational or communicative factors	Lack of support from relatives	**X**	**X**	**X**	Support from relatives or clinicians	**X**	**X**	**X**
					To share or not to share the experience	**X**	**X**	**X**
	To risk the patient–clinician relationship			**X**	Recognition from the clinicians	**X**		**X**
	Smoking is a sensitive topic			**X**	Clear messages	**X**	**X**	
					Non-judgmental approach	**X**	**X**	**X**
	Assumptions of patients’ knowledge of the risk of smoking			**X**	Understanding the link between smoking and bladder cancer	**X**	**X**	**X**
	Unstructured conversations in the clinical context			**X**	The smell of smoke is a facilitator for discussing smoking			**X**
					Mandatory to inform about smoking and bladder cancer			**X**

**X**: Represented in one or more interviews in the group. NRT: nicotine replacement therapy.

**Table 4:** Categories and sub-categories for recommendations. Patients have directly experienced the GSP, while relatives have encountered it second-hand. Clinicians, meanwhile, expressed their attitudes and expectations regarding the patient experience.

Categories	Sub-Categories	Patients	Relatives	Clinicians
**The GSP**	Participants recommended the provision of smoking cessation programmes in connection with the TURBT treatment.	**X**	**X**	**X**
	Participants recommended tailoring the smoking cessation intervention according to the patient’s treatment journey and symptom burden.	**X**		
	Participants suggested mandatory participation in a smoking cessation intervention in relation to TURBT treatment.	**X**		
	Participants recommended increasing the emphasis on the link between smoking and bladder cancer and its overall impact on health.	**X**		
	Participants recommended that the programme should increase the emphasis on the rapid health benefits of quitting smoking.	**X**		
	Participants recommended involving family members in the programme to let them share their pride and joy.		**X**	
	Participants recommended that NRT be offered free of charge for a longer period.	**X**		
**Pragmatic factors**	Participants recommended flexible programme options			**X**
	Participants recommended the provision of smoking cessation, even in patients who lack interest.			**X**
	Participants recommended that the smoking cessation intervention should be offered to smokers that might not actively ask for it.	**X**		
	Participants recommended a proactive approach to discussing smoking in clinical practice.			**X**
	Participants recommended the option of phone or video meetings to solve logistical challenges.			**X**
	Participants recommended electronic solutions to nudge towards initiating conversations with patients about smoking.			**X**
	Participants recommended the implementation of simple and efficient referral methods.			**X**
	Participants recommended training of clinicians’ skills in conversations about lifestyle changes.			**X**

**X**: Represented in one or more interviews in the group. Notes: NRT: nicotine replacement therapy.

## Data Availability

The datasets presented in this article are not readily available as they contain interview data which include personally identifiable information that cannot be shared to ensure compliance with the data protection regulation and to maintain the confidentiality of the participants.

## Abbreviations

The following abbreviations are used in this manuscript:

GSP	Gold Standard Programme
NRT	nicotine replacement therapy
SCI	smoking cessation intervention
TURBT	transurethral resection of the bladder tumour
